# Persian cats under first opinion veterinary care in the UK: demography, mortality and disorders

**DOI:** 10.1038/s41598-019-49317-4

**Published:** 2019-09-17

**Authors:** Dan G. O’Neill, Charlotte Romans, Dave C. Brodbelt, David B. Church, Petra Černá, Danièlle A. Gunn-Moore

**Affiliations:** 1 Pathobiology and Population Sciences, The Royal Veterinary College, Hawkshead Lane, North Mymms, Hatfield, Herts AL9 7TA UK; 2 Clinical Sciences and Services, The Royal Veterinary College, Hawkshead Lane, North Mymms, Hatfield, Herts AL9 7TA UK; 3 University of Veterinary and Pharmaceutical Sciences Brno, Palackého tř. 1946/1, 612 42 Brno, Czech Republic; 4 The Royal (Dick) School of Veterinary Studies and The Roslin Institute, University of Edinburgh, Easter Bush Campus, Midlothian, EH25 9RG UK

**Keywords:** Physiology, Dental diseases

## Abstract

Persian cats are a popular cat breed worldwide, and especially in the US, Europe and Asia. This study aimed to describe the demography, common disorders and mortality in Persians under general practice veterinary care in 2013 in the UK. The study population of 285,547 cats overall included 3235 (1.1%) Persians. Mean adult Persian bodyweight was 3.9 kg (SD 0.9) and median age was 7.0 years (IQR 3.3–11.6). At least one disorder was recorded in 2099 (64.9%) Persians. The most common specific disorders were haircoat disorders (411, 12.7%), periodontal disease (365, 11.3%), overgrown nails (234, 7.2%), and ocular discharge (188, 5.8%). The most common disorder groups were dermatological (578, 17.9%), ophthalmological (496, 15.3%) and dental (397, 12.3%). Median longevity was 13.5 years (IQR 9.9–16.0). The most common grouped causes of death were renal disease (102, 23.4%), neoplasia (37, 8.5%) and mass-associated disorder (35, 8.0%). This is the first study to use general practice data to examine the overall health of Persian cats. With haircoat, ocular and dental disorders being the predominant disorders identified, this study highlights the need for increased owner awareness to manage and prevent the typical health problems associated with this breed’s phenotype.

## Introduction

Domestic cats are popular pets in many countries, with the top 10 cat owning countries being the US (75–85 million cats), China (53 million), Russia (~13 million), Brazil (12.5 million), France (9.5 million), Italy (9.5 million), UK (8–11 million), Germany (~8 million), Ukraine (7.5 million), and Japan (~7 million)^1–3^. Purebred cats are popular in many countries, accounting for 16–18% of pet cats in the US^1^, and 8–11% of pet cats in the UK^4,5^. The Persian is one of the oldest cat breeds and was exhibited at a cat show for the first time in 1871, in Crystal Palace, London^6^. Persian cats are popular globally, being in the top five most numerous pedigree cat breeds and/or making up at least 5% of all pedigree kittens registered in 2017 in the US, much of Europe (particularly Italy, Spain, France, Norway, and the UK), plus many Asian countries (notably China and Japan)^7,8^. Persians are currently the second most popular cat breed in the US (after the Exotic) and the UK (after the British Short Hair)^5,7^. These data clearly demonstrate the popularity of Persian cats, and hence the need to understand their health risks and diseases more fully. In the UK alone, Persian cats account for around 1% of pet cats under primary veterinary care; equating to 100,000 pet cats in the UK currently^9^. Persian cats have many colour variants, including solid (such as blue, black and red), tortoiseshell and tabby, as well as these in combination with white. Himalayan (colour-point), golden and silver colour variants also referred to as Chinchilla. Exotics are the shorthair sister breed of Persian cats^10–13^.

Breed standards currently describe Persians as brachycephalic types that have a massive round head, great broad skull, short broad nose with a high nose-leather and the transition from nasal to frontal bone should form a “break” (“stop”) between the eyes^13–16^. However, while all Persians are considered brachycephalic, the degree of brachycephaly varies from breed lines that are more severely affected (e.g. Peke-faced or Ultra-type Persians) to some less extreme lines (e.g. Doll-faced, Open-type, or Classic Persians)^17^. Brachycephaly is important because the Persian skull shape has been associated with ophthalmic, facial, dental, respiratory, neurological and reproductive problems, and these conditions may be more severe in cats with more extreme brachycephaly^17,18^.

Despite their popularity, Persians are reportedly predisposed to 29 diseases to date, many of which are associated with their conformation or genotype^19^. Ophthalmic problems are reportedly common in Persians because of their large flat eye sockets and brachycephalic conformation. Reported ophthalmic conditions include chronic epiphora related to kinking of the nasolacrimal ducts that can result in facial irritation^18,20^, non-healing corneal ulcers^21^, corneal sequestra^22^, entropion^23^ and decreased corneal sensitivity compared with non-pedigree cats^24^. Persians are also reported with ophthalmic disorders with a known genetic cause, including an early-onset, autosomal recessive form of progressive retinal atrophy. This disease often begins early in life, before 3 weeks of age, and an progress rapidly with affected cats showing marked photoreceptor loss by 15 weeks of age^25^.

Persian cats are reported with Brachycephalic Obstructive Airway Syndrome (BOAS), as are brachycephalic dog breeds^26^, with stenotic nares, compressed nasal turbinate bones and retrograde conchae leading to obstruction of the ventral nasal passage^17^, as well as respiratory dysfunction associated with reduced muzzle length^18^ and, occasionally, elongated soft palate^27^. In contrast to brachycephalic dogs^28^, brachycephalic cats are not reported with reduced tracheal diameter^29^.

The brachycephalic skull conformation also predisposes Persians to cerebellar crowding and herniation compared with mesocephalic breeds^30^. Persian kittens with extreme brachycephaly (i.e. Peke-face Persians) have increased risk of hydrocephalus and associated neurological defects, deafness, pain, severe mental retardation, and death^17^. Less severely affected kittens are uninterested in interacting with their owners or other cats^17^. Of note, two of the genes linked with being Persian (*CHL1* and *CHL6*) are associated with mental retardation and autism in people^31^. The malformed facial bones can cause dental disarray, causing Persians to have problems prehending food and leading to a higher risk of secondary dental disease, most notably malocclusion and/or crowding of the teeth^17,32^.

Persians are more likely to develop dystocia than mesocephalic cats because the relatively large brachycephalic foetal skull has difficultly passing through the narrow maternal pelvic canal typical of the breed, resulting in foetal malposition and uterine inertia^33,34^. Dystocia frequently results in stillbirth, resulting in a higher mean stillbirth rate in Persians of 11% compared to 8.2% across all purebred cats, and giving Persians the highest kitten mortality rate of all purebred cats, at 25%^35^.

The Persian breed is described as having a short sturdy body and short thick legs, and a dense long haircoat^13–16^. Compared to other breeds, Persians have an increased risk of coat and skin problems, including dermatophyte infections and pseudomycetomas, which may be associated with their dense and long coats^36,37^. Moreover, Persian cats appear to be genetically predisposed to dermatophytosis^38–40^ possibly because they have two copies of the mutated gene version for fibroblast growth factor 5 which causes abnormally long hair growth making them more likely suffer from this disease^41,42^, and may also involve a defect in genetic cell-mediated immunity that is linked with inheritance of the gene for long hair and may also be associated with a predisposition to dermatophytosis, although the full causal pathway remains to be elucidated^43–45^. Dermatological conditions may be compounded by hampered ability to groom related to other conditions such as dental disease^17,46,47^, arthritis^48,49^, or other causes of pain^50^.

Persians are reported as predisposed to diseases of the urinary system^51^ including autosomal dominant polycystic kidney disease (ADPKD)^52^, urolithiasis, and congenital defects affecting the bladder^53,54^. ADPKD is one of the most prominent inherited diseases of Persian cats^55^. It had a prevalence of 49% in the UK between 1996 and 2000; however, that value was calculated from only 132 high-risk Persians^52,56^. Because ADPKD can lead to renal failure, cat societies such as the Governing Council of the Cat Fancy require breeding Persians to be screened for the ADPKD gene defect^13^. Langford Veterinary Diagnostics have documented a decreasing prevalence of the ADPKD gene in Persians in the UK, from ~28% in 2005 to ~2% in 2016^57^, showing how effective this type of targeted intervention can be.

Given the breadth of conditions that have been reported as over-represented in Persians across geographical locations, breed lines and time, there is a need for accurate, generalisable and recent data on the frequencies and types of common disorders in this breed, to provide guidance for breeding, clinical prioritisation and research^58,59^. Using anonymised clinical data from the VetCompass Programme^60^, this study aimed to report on the demography, common disorders and mortality of a large population of Persian cats under general practice veterinary care in the UK. Special focus was placed on exploring associations with haircoat and sex.

## Results

### Demography

**Figure 1:**
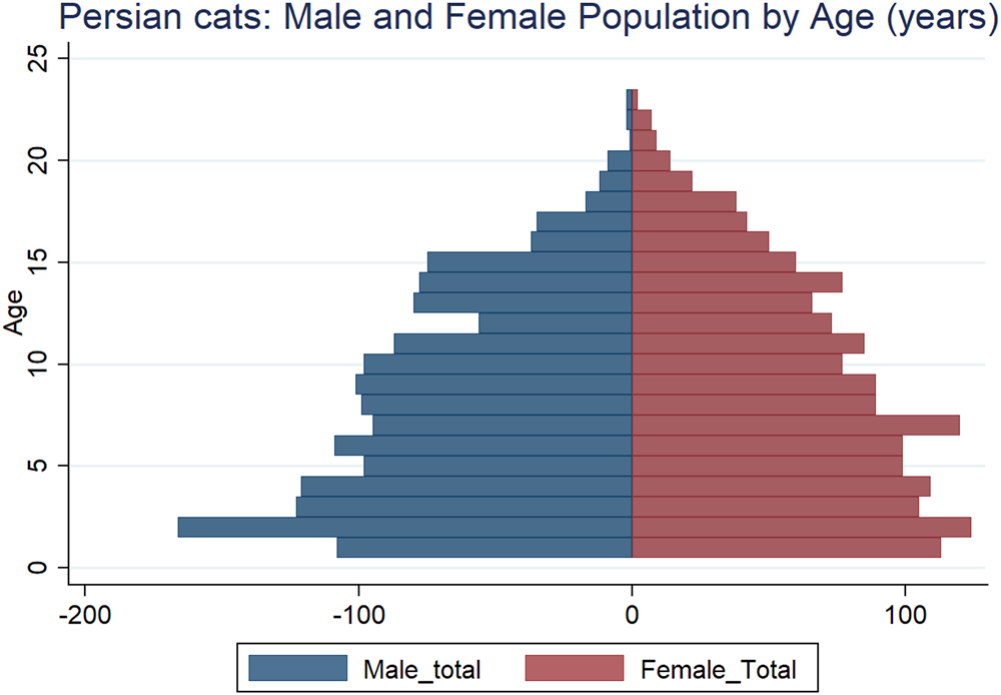
Age pyramid for female and male Persian cats attending UK general-care veterinary clinics participating in the VetCompass Programme. (Females *n* = 1569, Males *n* = 1609).

**Figure 2:**
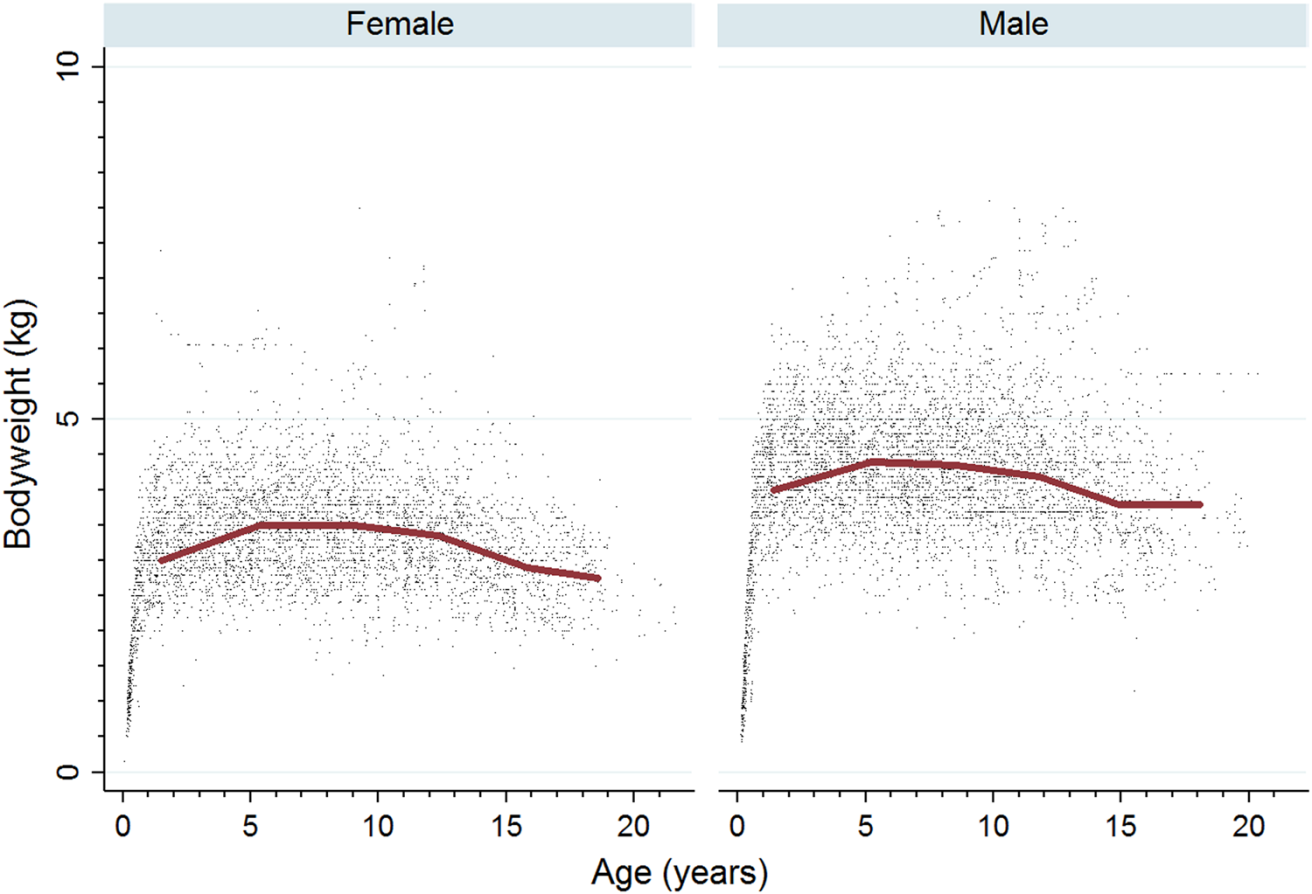
Bodyweight growth curves overlaid with a cross medians line plot for female and male Persian cats attending UK general-care veterinary clinics participating in the VetCompass Programme. (Females *n* = 909, Males *n* = 951).

The study population of 285,547 cats came from 304 clinics in the VetCompass database under veterinary care during 2013, and included 3,235 (1.13%) Persians. Of Persians with information available, 1,596 (49.6%) were female and 1,921 (77.0%) were neutered. Males were more likely to be neutered than females (79.9% versus 75.2%, *P* = 0.006). The median age was 7.0 years (IQR 3.3–11.6, range 0.1–24.7) (Fig. 1). The mean adult bodyweight was 3.9 kg (standard deviation [SD] 0.9). Adult males (4.3 kg, SD 0.8 kg) were heavier than females (3.4 kg, SD 0.7 kg) (*P* < 0.001) (Table 1). Bodyweight growth curves based on 5,039 bodyweight values from 909 females and 5,762 bodyweight values from 951 males showed that Persians grow rapidly during their first year but that males plateau at a higher adult bodyweight than females, before both sexes gradually drop weight beyond 12 years of age (Fig. 2). The median bodyweight across all ages for males (4.2 kg, IQR: 3.7–4.8, range: 0.4–8.1) was higher than for females (3.3 kg, IQR: 2.8–3.8, range: 0.2–8.0) (P < 0.001). Data completeness varied across the variables assessed: sex 99.4%, age 98.7%, neuter 77.1% and all-age bodyweight 57.8%. The relatively low level of completeness for neuter status in these data should be borne in mind when interpreting results based on neuter status because of high risks of selection bias for the status recorded.

**Table 1:** Demography of Persian cats under general practice veterinary care at clinics participating in the VetCompass Programme in the UK from January 1^st^, 2013 to December 31^st^, 2013 (n = 3,235).

Variable	Category	Count*	Percent
Sex	Female	1,596	49.6
	Male	1,619	50.4
Female neuter	Entire	303	24.8
	Neutered	920	75.2
Male neuter	Entire	252	20.1
	Neutered	1,000	79.9
Female adult bodyweight (aged ≥1 year) (kg)	1.0 to <3.0	249	29.5
	3.0 to <4.0	439	52.1
	4.0 to <5.0	138	16.4
	≥5.0	17	2.0
Male adult bodyweight (aged ≥1 year) (kg)	1.0 to <3.0	41	4.6
	3.0 to <4.0	281	31.8
	4.0 to <5.0	402	45.4
	≥5.0	161	18.2
Age (years)	0.0 to <3.0	741	23.2
	3.0 to <6.0	637	20.0
	6.0 to <9.0	597	18.7
	9.0 to <12.0	475	14.9
	12.0 to <15.0	435	13.6
	≥15.0	307	9.6

*Count covers cats with available data.

### Disorder prevalence

Of the 3,235 Persians in the study, 2,099 (64.9%) had at least one disorder recorded during 2013 while the remaining 35.1% had no disorder recorded and either presented for prophylactic management only or did not present at all during 2013. The median count of disorders was 1 (IQR 0–2, range 0–15) and did not differ between females (median 1, IQR 0–2, range 0–10) and males (median 1, IQR 0–2, range 0–15) (*P* = 0.620).

The study included 5330 unique disorder events recorded during 2013, encompassing 368 distinct fine-level disorder terms. The most prevalent fine-level precision disorders were haircoat disorder (n = 411, prevalence 12.7%, 95% CI: 11.6–13.9), periodontal disease (365, 11.3%, 95% CI: 10.2–12.4), overgrown nails (233, 7.2%, 95% CI 6.3–8.1) and ocular discharge (188, 5.8%, 95% CI 5.0–6.7). Among the 25 most common fine-level precision disorders, males were more likely than females to be diagnosed with periodontal disease (12.6% versus 10.0% respectively, *P* = 0.018) and obesity (2.6% versus 0.9% respectively, *P* < 0.001). Conversely, females were more likely than males to be diagnosed with overgrown nails (8.5% versus 6.1% respectively, *P* = 0.009) and anorexia/not eating (4.7% versus 2.8% respectively, *P* = 0.006) (Table 2). ADPKD was recorded in 22 cats (prevalence 0.7%, 95% CI: 0.4–1.0).

**Table 2:** Prevalence of the most common disorders at a fine-level of diagnostic precision recorded in Persian cats (n = 3,235) attending UK general practice veterinary clinics participating in the VetCompass Programme from January 1^st^, 2013 to December 31^st^, 2013.

	Fine-level disorder	Count	Overall prevalence %	95% CI*	Female prevalence %	Male prevalence %	P-Value
1	Haircoat disorder	411	12.7	11.6–13.9	13.1	12.5	0.600
2	Periodontal disease	365	11.3	10.2–12.4	10.0	12.6	**0.018**
3	Overgrown nail(s)	233	7.2	6.3–8.1	8.5	6.1	**0.009**
4	Ocular discharge	188	5.8	5.0–6.7	5.5	6.2	0.380
5	Heart murmur	188	5.8	5.0–6.7	5.1	6.6	0.064
6	Fleas infestation	165	5.1	4.4–5.9	5.3	5.0	0.738
7	Weight loss	160	4.9	4.2–5.8	4.7	5.2	0.522
8	Conjunctivitis	146	4.5	3.8–5.3	4.3	4.8	0.499
9	Diarrhoea	142	4.4	3.7–5.2	4.8	4.0	0.264
10	Vomiting	140	4.3	3.7–5.1	3.6	4.9	0.067
11	Hyporexic/anorexic	121	3.7	3.1–4.5	4.7	2.8	**0.006**
12	Chronic renal failure	116	3.6	3.0–4.3	3.9	3.3	0.404
13	Underweight	102	3.2	2.6–3.8	3.6	2.7	0.135
14	Otitis externa	81	2.5	2.0–3.1	2.4	2.6	0.785
15	Sneezing	80	2.5	2.0–3.1	2.2	2.8	0.286
16	Tear duct abnormality	76	2.3	1.9–2.9	2.3	2.5	0.688
17	Ulcerative keratitis	76	2.3	1.9–2.9	2.1	2.7	0.272
18	Polyuria/polydipsia	66	2.0	1.6–2.6	2.2	1.9	0.578
19	Corneal disorder	61	1.9	1.4–2.4	1.6	2.2	0.172
20	Cystitis	57	1.8	1.3–2.3	2.0	1.5	0.322
21	Obesity	57	1.8	1.3–2.3	0.9	2.6	<**0.001**
22	Nasal discharge	56	1.7	1.3–2.2	1.8	1.6	0.644
23	Upper respiratory tract disorder	52	1.6	1.2–2.1	1.6	1.7	0.820
24	Upper respiratory tract infection	50	1.5	1.1–2.0	1.2	1.9	0.097
25	Trichobezoar (hairball)	47	1.5	1.1–1.9	1.3	1.5	0.586

The P-value reflects prevalence comparison between females and males. *CI confidence interval.

Haircoat disorder (n = 411) and periodontal disease (n = 365) were each associated with a differing subset of 12 of the 24 other most common fine-level precision disorders (Table 3).

**Table 3:** Associations between cats with haircoat disorder (n = 411) and periodontal disease (n = 365) with the other most common fine-level precision disorders recorded in Persian cats (n = 3,235) attending UK general practice veterinary clinics participating in the VetCompass Programme from January 1^st^, 2013 to December 31^st^, 2013.

	Fine-level disorder	Count (%) of 2,824 cats without haircoat disorder	Count (%) of 411 cats with haircoat disorder	P-Value	Count (%) of 2,870 cats without periodontal disorder	Count (%) of 365 cats with periodontal disorder	P-Value
1	Haircoat disorder	~	~	**~**	336 (11.7)	75 (20.6)	<**0.001**
2	Periodontal disease	290 (10.3)	75 (18.3)	<**0.001**	~	~	~
3	Overgrown nail(s)	161 (5.7)	72 (17.5)	<**0.001**	195 (6.8)	38 (10.4)	**0.012**
4	Ocular discharge	156 (5.5)	32 (7.8)	0.067	148 (5.2)	40 (11.0)	<**0.001**
5	Heart murmur	145 (5.1)	43 (10.5)	<**0.001**	147 (5.1)	41 (11.2)	<**0.001**
6	Fleas infestation	129 (4.6)	36 (8.8)	<**0.001**	140 (4.9)	25 (6.9)	0.107
7	Weight loss	128 (4.5)	32 (7.8)	**0.004**	130 (4.5)	30 (8.2)	**0.002**
8	Conjunctivitis	129 (4.6)	17 (4.1)	0.694	121 (4.2)	25 (6.9)	**0.022**
9	Diarrhoea	108 (3.8)	34 (8.3)	<**0.001**	123 (4.3)	19 (5.2)	0.419
10	Vomiting	116 (4.1)	24 (5.8)	0.107	111 (3.9)	29 (8.0)	<**0.001**
11	Anorexia/not eating	90 (3.2)	31 (7.4)	<**0.001**	105 (3.7)	16 (4.4)	0.492
12	Chronic renal failure	94 (3.3)	22 (5.4)	**0.039**	98 (3.4)	18 (4.9)	0.142
13	Underweight	128 (4.5)	32 (7.8)	**0.004**	89 (3.1)	13 (3.6)	0.635
14	Otitis externa	63 (2.2)	18 (4.4)	**0.009**	65 (2.3)	16 (4.4)	**0.015**
15	Sneezing	66 (2.3)	14 (3.4)	0.192	69 (2.4)	11 (3.0)	0.480
16	Tear duct abnormality	62 92.2)	14 (3.4)	0.130	61 (2.1)	15 (4.1)	**0.018**
17	Ulcerative keratitis	64 (2.3)	12 (2.9)	0.414	63 (2.2)	13 (3.6)	0.104
18	Polyuria/polydipsia	48 (1.7)	18 (4.4)	<**0.001**	55 (1.9)	11 (3.0)	0.162
19	Corneal disorder	48 (1.7)	13 (3.2)	**0.042**	46 (1.6)	15 (4.1)	**0.001**
20	Cystitis	48 (1.7)	9 (2.2)	0.480	45 (1.6)	12 (3.3)	**0.019**
21	Obesity	48 (1.7)	9 (2.2)	0.480	44 (1.5)	13 (3.4)	**0.006**
22	Nasal discharge	47 (1.7)	9 (2.2)	0.445	51 (1.8)	5 (1.4)	0.574
23	Upper respiratory tract disorder	42 (1.5)	10 (2.4)	0.154	42 (1.5)	10 (2.7)	0.068
24	Upper respiratory tract infection	44 (1.6)	6 (1.5)	0.880	45 (1.6)	5 (1.4)	0.773
25	Trichobezoar (hairball)	39 (1.4)	8 (2.0)	0.371	39 (1.4)	8 (2.2)	0.210

The study included 4866 unique grouped-level precision disorder events recorded during 2013 that encompassed 56 distinct grouped-level precision disorder terms. The most prevalent grouped-level precision disorders were dermatological/coat (n = 578, prevalence: 17.9%, 95% CI 16.6–19.2), ophthalmological (496, 15.3%, 95% CI: 14.1–16.6) and dental (397, 12.3%, 95% CI: 11.1–13.5). Among the 15 most common grouped-level precision disorders, males were more likely than females to be diagnosed with a dental disorder (13.8% versus 10.7% respectively, P = 0.007), whereas females were more likely than males to be diagnosed with a claw/nail disorder (8.7% versus 6.3% respectively, *P* = 0.011) and a reduced appetite disorder (5.0% versus 3.1% respectively, *P* = 0.006) (Table 4).

**Table 4:** Prevalence of the most common grouped-level disorders recorded in Persian cats (n = 3,235) attending UK general practice veterinary clinics participating in the VetCompass Programme from January 1^st^, 2013 to December 31^st^, 2013.

Grouped-level disorder	Count	Overall prevalence %	95% CI*	Female prevalence %	Male prevalence %	P-Value
Cutaneous	578	17.9	16.6–19.2	18.4	17.6	0.577
Ophthalmological	496	15.3	14.1–16.6	15.3	15.4	0.943
Dental	397	12.3	11.1–13.5	10.7	13.8	**0.007**
Enteropathy	374	11.6	10.5–12.7	11.4	11.7	0.768
Underweight	264	8.2	7.2–9.2	8.3	7.9	0.657
Upper respiratory tract	242	7.5	6.6–8.4	6.8	8.2	0.154
Claw/nail	240	7.4	6.5–8.4	8.7	6.3	0.011
Cardiac	229	7.1	6.2–8.0	6.0	8.2	0.015
Parasitic	207	6.4	5.6–7.3	6.5	6.4	0.972
Renal	174	5.4	4.6–6.2	5.5	5.3	0.861
Urinary system	163	5.0	4.3–5.8	5.6	4.6	0.194
Reduced appetite	131	4.0	3.4–4.8	5.0	3.1	**0.006**
Aural	112	3.5	2.9–4.2	3.5	3.5	0.908
Musculoskeletal	91	2.8	2.3–3.4	2.6	3.0	0.432
Mass-associated	89	2.8	2.2–3.4	2.6	2.9	0.562

The P-value reflects prevalence comparison between females and males. *CI confidence interval.

### Mortality

There were 575 deaths recorded during the study. The median age at death (longevity) of Persians overall was 13.5 years (IQR 9.9–16.0, range 0.0–24.0). Females (14.0 years, IQR 10.3–16.7, range 0.0–22.6, n = 298) lived longer than males (13.1 years, IQR 9.2–15.6, range 0.2–24.0, n = 271) (*P* = 0.003). For cats that died after one year of age, there was no difference in median longevity between entire animals (13.9 years, IQR 12.0–15.8, range 3.0–21.0, n = 62) and neutered animals (13.4 years, IQR 10.0–16.1, range 1.3–23.6, n = 395) (*P* = 0.455). Of the 435 (75.6%) cats where cause of death was stated, the most common causes at a grouped-precision level were renal disease (n = 102, 23.4%), neoplasia (37, 8.5%), and mass-associated disorder (35, 8.0%) (Table 5). Euthanasia accounted for 454/538 (84.4%) death, where the mechanism was recorded while 84 (15.6%) were unassisted.

**Table 5:** Mortality in Persians with a recorded cause of death under general practice veterinary care at UK clinics participating in the VetCompass Programme from January 1^st^, 2013 to December 31^st^, 2013 (n = 435).

Grouped-level disorder	Overall Count (%)	Female count (%)	Male Count (%)	P-Value male vs female
Renal disease	102 (23.4)	54 (23.1)	48 (24.1)	0.114
Neoplasia	37 (8.5)	16 (6.8)	21 (10.6)	0.384
Mass-associated disorder	35 (8.0)	19 (8.1)	16 (8.0)	0.398
Collapsed	27 (6.2)	14 (6.0)	13 (6.5)	0.463
Appetite-assocaited	25 (5.7)	16 (6.8)	9 (4.5)	0.481
Thinness	21 (4.8)	13 (5.6)	8 (4.0)	0.520
Traumatic injury	19 (4.4)	9 (3.8)	10 (5.0)	0.542
Brain disorder	16 (3.7)	9 (3.8)	7 (3.5)	0.577
Heart disease	14 (3.2)	7 (3.0)	7 (3.5)	0.603
Spinal cord disease	13 (3.0)	8 (3.4)	5 (2.5)	0.616
Other	126 (29.0)			
Total	435 (100.0)			

The P-value reflects comparison between the prevalence in females and males.

## Discussion

To date, this is the largest study of breed-related health in Persian cats; in this case using a general practice veterinary population. The findings are of particular significance given the global popularity of domestic cats as pets, and of the Persian breed specifically. Haircoat problems, periodontal disease, overgrown nails, and ocular discharge were the most common specific disorders diagnosed, whilst cutaneous, ophthalmological and dental disease were the most common disorder groups. These results provide veterinarians with a strong evidence-base to prioritise advice given to owners on preventive care in Persians, which could markedly improve Persian cat health and welfare^5^. These results for a cat breed with extreme brachycephaly will also support ongoing efforts to understand the implications of brachycephaly for other companion species, such as the dog, where there is currently substantial debate on the health and welfare impacts of this facial feature^61,62^.

Most previous studies concerning Persians were based on smaller referral caseloads and/or focused on specific diseases^20,24,52,63,64^, rather than examining the prevalence of disorders more generally in the wider cat population. Although useful for referral clinicians and possibly for studies of pathogenic pathways in severely affects subsets of diseases, referral studies offer limited extrapolation to the wider caseload seen in general practice^65^. The use of such a large, general practice-derived data set, as interrogated in the current study, gives statistical power and generalisability to our results. However, failure to follow many cases through to a definitive diagnosis can result in the primary-care methodology losing some fine-level diagnostic granularity to assess specific disease conditions that may be more readily available in more focused referral studies^65,66^. In summary, stronger epidemiological inference can be taken when results from several methodologies are considered^65^.

Haircoat disorders were the most prevalent fine-level diagnosis, affecting 12.7% of the Persian cats in this study. For comparison, only 2.5% of non-purebred cats in another VetCompass study had coat disorders, although this rose to 5.6% in purebreds^5^. When ordered by disorder group, cutaneous disorders were also the most common group, affecting 17.9% of the Persians. These findings agree with a US referral study that also showed a predisposition to dermatological disorders in Persians and suggests important welfare implications from discomfort, and repeated sedations for grooming, de-matting and their related injuries^36^. It is likely that Persians may also have an inherently reduced grooming ability resulting from their brachycephalic conformation^17,67^; however, no breed-comparative studies have yet been conducted to confirm this.

Inherent predisposition to haircoat problems may be complicated by interactions with other conditions prevalent in the breed. The current study showed positive associations between haircoat disorders and 12 of the 24 other common fine-level diagnoses suggesting that haircoat problems are closely interlinked with the overall health of Persian cats. Some of the associated conditions included periodontal disease (which is of particular note as it and ocular disease were the most common problems in this breed, and all three relate to the breed phenotype); chronic renal failure and polyuria/polydipsia (which is not surprising since renal disease was the most common cause of death in these cats); overgrown nail(s), otitis externa, and flea infestation (suggesting poor husbandry where owners find caring for these long-haired cats challenging); weight loss, anorexia/not eating, and being underweight (which are likely all related); plus corneal disorder. The association with corneal disorder is of particular note as it is the only one of the 5 common ocular disorders where an association with haircoat problems was found. This suggests weak (or no) health links between the 2 most striking phenotypes of being a Persian cat (i.e. having a long dense haircoat and brachycephaly-associated ocular disorders).

It should be noted that association does not specify a direction of effect or even imply any causality^68^. Consequently, for example, it may be that periodontal disease causes dental pain that leads to reduced grooming behaviour or it could be that the prolonged grooming promotes periodontal disease^47^ or, indeed potentially, that the association was confounded by another condition. Grooming is a major activity in cats, with domestic cats spending up to 24% of their waking time grooming^69^ in order to control parasites, remove loose hairs and for thermoregulation^70–72^. However, grooming is challenging for cats because of their two layers of fur: an exposed topcoat for protection covers a hidden undercoat of down hairs for warmth^73^. A recent study of grooming kinematics explored grooming activity across a range of cat species and breeds to show that the long and dense coats of Persian cats prevents full access to the deeper layers of their fur and make these Persian coats ‘ungroomable’^74^.

The associations shown in the current study encourage us to recommend that when veterinarians encounter matted coats in Persians they should evaluate these patients carefully for dental disease, as these are likely to be comorbid conditions. As indicated above, the particular characteristics of the Persian coat makes it especially difficult to groom; it is also possible that extrinsic factors such as the amount of grooming the cat receives from its owner influences the occurrence of coat problems. Difficulty grooming can mean that faecal material can gathers on a Persian cat’s perineum which, in warm countries, can promote cutaneous myiasis (fly strike) that can be fatal if not promptly addressed^75^. This underlines the importance of veterinarians providing advice to Persian cat owners about routine grooming and its importance for their cat’s health.

Brachycephalic dogs have been reported with significantly more ear disease than mesocephalic breeds (particularly associated with primary secretory otitis media)^76^. It is therefore of note that the current study found 2.5% of Persians with otitis externa, while this was not in the top 20 conditions seen in the overall cat population^5^. Although a direct Persian versus non-Persian comparison was not made in the current study, Persians may be predisposed to otitis externa because of excessive ceruminous gland production^77^.

Ophthalmological problems were the second most common group of disorders, occurring in 15.3% of Persians, compared to a previous report of just 6.7% of the general population, and 9.3% in purebred cats^5^, confirming the predisposition of Persians to ocular disease overall^46,51,54^. Of the 5/25 specific ocular disorders listed on Table 2, only conjunctivitis featured within the top 20 conditions seen in the overall cat population^5^. Of note, of these 5 specific ophthalmological disorders recorded [ocular discharge (5.8%), conjunctivitis (4.5%), tear duct abnormalities (2.3%), ulcerative keratitis (2.3%) and corneal disorder (1.9%)], only ocular discharge was ranked in the top 5 specific disorders in the current study; this illustrates the importance of multi-level hierarchy analyses when interpreting the results of all-diagnosis studies to avoid omitting crucial summative findings^78^.

Other authors have reported an increased occurrence of ocular discharge in Persians^79^, and tear-staining is considered a common feature of the breed^6,18^, with Persian breed societies advocating routine eye bathing^13^. The brachycephalic facial conformation results in epiphora because nasolacrimal duct malformation reduces effective nasolacrimal drainage^20,80^, while the prominent globe reduces the depth of the lacrimal lake, so tears run down the face^80^. Persians also have a reduced ability to remove ocular irritants because of ineffective blinking and inappropriate tear production^81^, which predisposes to corneal ulceration and ulcerative keratitis^82^. Brachycephalic cats, like brachycephalic dogs, also show decreased corneal sensitivity compared to normal cats and, paradoxically, a reduced ocular pain response in brachycephalic cats means that owners may fail to recognise these problems, or to act on them quickly, which only compounds these issues^24,81,83,84^.

Conjunctivitis was recorded in 4.5% of Persians in the current study, compared with only 2.9% in all purebred cats and 3.0% of the overall cat population^5^, supporting previous reports of an increased risk of conjunctival inflammation in Persians^54^. Conjunctivitis has been associated with ocular defects, such as entropion^23^. However, this and other structural defects were not reported sufficiently frequently in the current study for them to be major causes of conjunctival inflammation in Persians. This implies that the conjunctival inflammation was likely due to ocular infection in most cases^85^. Since the prevalence of conjunctivitis in Persians was 4.5% in the current study, but only 2.9% in all purebred cats^5^, this suggests that Persians may have an intrinsically increased susceptibility to ocular infection. In contrast, this prevalence trend was not matched in upper respiratory tract (URT) disease, which is often caused by the same infectious agents as conjunctivitis^86,87^; URT infection was recorded in 7.5% of Persians in the current study, compared to 10.6% of all purebred cats^5^. This alludes to there being something specific about Persian eyes that makes them particularly susceptible to infection. Owners should be aware of these risks and veterinarians should pay special attention to the eyes of Persians whenever these cats are presented.

Dental disorders were the third most common grouped disorder (12.3%) and periodontal disease was the second most common specific disorder recorded (11.3%), clearly marking out dental health as a priority for Persian cat management. However, periodontal disease is a common condition across all cat breeds. A similar study reported dental disease in 13.9% of pet cats in the UK^5^. The high prevalence of dental disease in Persians, regardless of the absence of a predisposition in the breed, suggest that veterinarians could reasonably improve their promotion of preventative periodontal care^88,89^. Evidence for Persian (and Exotic) cats having an increased predisposition of certain dental disorders comes from a recent prospective study where 50 cats were anaesthetized for complete dental examinations and identified 88% with periodontal disease, 76% with abnormal numbers of teeth, 72% with dental malocclusions, 64% with malpositioned teeth and 56% with dental crowding^32^. The current retrospective study relied on typical general practice clinical care and identified dental problems in only 12.3% of the cats which suggests, compared to this recent prospective study^32^, that dental disease may have been substantially under-reported in the present study. Of note, periodontal disease in Persians may be associated with other conditions prevalent in this breed. The current study found positive associations between periodontal disease and 4 of the 5 common fine-level ophthalmological disorders (ocular discharge, conjunctivitis, tear duct abnormality, and corneal disorder). That the study demonstrated a positive association between periodontal disorders and ophthalmological disorders in Persians is important; these problems may arise concomitantly because they are both predisposed to by the facial deformities that accompany brachycephaly.

Published literature shows Persian cats are predisposed to respiratory^18,27^, reproductive^33,35^, and neurological^17,30^ disorders. However, the current study failed to find evidence to support these prior reports. While URT disease was reported in 7.7% of Persians in the current study, it was more prevalent in all pedigree cats (10.7%) than non-pedigrees (4%)^5^, making a particular breed predisposition difficult to determine. In addition, some owners of brachycephalic dogs think their pets’ noisy breathing is ‘normal for their breed’^90^. If owners of Persian cats think the same way, some cases of URT disease may never have been presented to a veterinarian in our study, so leading to reduced recognition. Reproductive disorders were not reported in the top 20 disorders of Persian cats in the current study, nor in non-pedigree cats^5^. However, the lack of recognition of reproductive problems is not surprising since few of the 1200 apparently entire female Persians in the current study would have been bred in a single year, and the study was not designed with the power to specifically explore disorders with under 2% expected prevalence. Neurological disorders were not reported in either the fine or grouped level diagnostic disorders in the Persians in the current study, nor in non-pedigrees^5^. However, owners of Persian cats may not expect this breed to be particularly interactive, so cats less severely affected by cerebellar herniation and hydrocephalus may again be assumed as ‘normal for breed’^17,30^. Brain disorder was given as the cause of death in 3.7% of the Persians, compared to 2% of all cats in Sweden and 15% of all cats in the UK^4,51^. Unfortunately, the lack of detail in these studies means it is not possible to determine the age of these neurological cases, nor the nature of their neurological disease.

While the most significant findings in the current study were found comparing Persians with other breeds, and/or non-purebred cats, there were also a number of notable sex-related findings. Males were more likely than females to be neutered, presumably because fewer males are needed for breeding. Males were also more likely to be overweight/obese and to have periodontal disease. In contrast, females were more likely to be hyporexic/anorexic and to have nail problems.

The leading cause of mortality in Persians in the current study (at or after 5 years of age) was renal disease (23.4%). The high impact of renal disease as a cause of death in Persians agrees with a study of insured cats in Sweden^51^. An earlier VetCompass study into longevity in the general cat population in UK also identified renal disease as the most common cause of death in cats at or older than 5 years; however, the prevalence was just 13.6% of these deaths^4^. While this suggests a predisposition to renal dysfunction in Persians, the underlying cause(s) remain unclear. Notably, ADPKD was recorded in just 22 cats (prevalence 0.7%) which supports the success of screening schemes in removing this genetic defect from the breed^57^. While it is possible that some cases of mortality resulting from ADPKD may have been missed in the current study, it is hoped that a high proportion of the true cases were clinically detected because of the obvious renomegaly that this disease typically causes^91^.

Neoplasia accounted for 8.5% of deaths in the current study, with a similar value of 8.0% for deaths from mass-associated disorder; these findings are similar to the proportions of deaths from these causes in the overall UK cat population^4^. It is worth considering that, while mass-associated disorders may also include infection and inflammation, many are likely to be unconfirmed neoplasia and it is probable that neoplastic disorders are under-diagnosed in general practice and may account for a greater proportion of deaths than currently specifically recorded.

The median age of death of 13.5 years for Persians in the current study was not substantially different to the 14.0 years previously reported in the UK general cat population^4^. This shows that while Persians may have a higher prevalence of dermatological and ocular ill-health than many other purebred or non-pedigree cats^5,46^, they can still live a long life.

This study had some limitations. The study included data from a single breed (Persian cats), which were compared in the discussion against results on other pet cats from other studies to expand inference. This inferential process is commonly used in publications for logistical reasons, although extraction and comparison of data across multiple breeds within a single study would provide stronger evidence^19^. The quality of EPR data relies on the detail and clarity of the clinical records kept by individual veterinarians, and EPR data were not recorded primarily for research purposes^65^. Many of the terms included as diagnosis terms were presenting signs (e.g. vomiting). This was especially the case where a full clinical work-up was not performed, either because symptomatic treatment was appropriate and/or due to other limiting factors (e.g. finance or lack of owner motivation), so not all disorders were definitively diagnosed to a precise aetiopathological cause. Additionally, typical of primary-care practice protocols, many of the final diagnoses recorded were at a general level and did not pursue diagnostic investigation to define highly precise diagnostic terms^66^. In contrast to the referral situation where determining a biomedical diagnosis is a core feature of the clinical management process, the primary-care clinician is focused on reaching a level of diagnostic insight that supports a treatment plan that is acceptable in terms of clinical, financial, temporal, welfare, owner preference and logistical outcome; such an outcome may not require or may even be hindered by an inflexible demand to define a final biomedical diagnosis term^92–94^. Neuter status was recorded for just 77.1% of Persians and, where present, likely reflected the cat’s status when first registered at the practice; it may not have been updated following neutering in all cases. VetCompass data collection uses an anonymized approach that is reliant on the quality and completeness of data entry by the originating veterinary practices and can be complicated by varying data entry strategies across the many computerized practice management systems currently in use in the UK. The relatively low level of completeness for neutering status could partially explain the unlikely finding that only 77% of Persians were neutered. It also questions the finding that neutered and un-neutered cats were statistically similar ages at death. A significant proportion of mortality data had no cause of death listed (24.6%), particularly where the owner reported their cat had died away from the practice, but gave no further explanation, so traumatic events may have been underestimated^4^. Disorders were ranked on their prevalence; however, to determine their true overall impact on welfare, additional information on duration and severity would be required^95^. It is also worth noting that this study applied multiple comparisons based on univariable analyses and therefore the findings should be interpreted with caution.

## Conclusions

Since Persian cats represent a significant proportion of the global pedigree cat population, this largest ever study into breed-related health and disease in Persian cats in a general practice population adds significantly to our understanding of the problems they may encounter, especially since it is already clear that these adverse effects increase with increasing severity of brachycephalia^17,18^. However, the authors do not believe that banning an entire breed is the best way to deal with this growing brachycephalia issue in cats. Impacts from the Dangerous Dogs Act^96^ in the UK are evidence of how breed-specific legislation can be poorly effective, and may wrongly target many healthy animals^97^. Similar mixed results have been reported in other countries^98^. The authors consider that it is better to work with all interested parties to educate breeders, veterinary surgeons, and the public about the health concerns from severe brachycephalia. If Breed Standards for Persians are updated, breeders of cats with less pronounced brachycephalia will start winning at shows and will become the new norm. Owners will demand kittens with less extreme brachycephalia and there will be a natural move towards less-brachycephalic types i.e. Doll-faced, Open-type, or Classic Persians and away from the extremely brachycephalic types i.e. Peke-faced or Ultra-type Persians. However, if there are not meaningful moves away from the more extreme aspects of facial conformation in Persian (and Exotic) cats within a reasonable period (perhaps 5 years), the authors accept that specific legislation may be necessary e.g. that breeding cats with the top of their nose leather at or above the bottom of the medial canthus of their eye would be deemed animal cruelty.

## Methods

The methods used in the current study are deliberately similar to the methods used in some other VetCompass breed-based studies to facilitate comparisons between breeds of cats and dogs^99–101^ The study population included all cats under general practice veterinary care at clinics participating in the VetCompass Programme during 2013. Cats were included in the study provided they had i) at least one electronic patient record [EPR] (VeNom diagnosis term, free-text clinical note, treatment or bodyweight) recorded during 2013, and/or ii) at least one EPR recorded *both* before and after 2013. The VetCompass Programme collates de-identified EPR data from primary-care veterinary practices in the UK for epidemiological research^60^. Collaborating practices can record summary diagnosis terms during episodes of care from an embedded VeNom Code list^102^. Data fields available for VetCompass researchers include a unique animal identifier along with species, breed, date of birth, sex, neuter status, bodyweight, free-form text clinical notes, VeNom summary diagnosis terms and treatment with relevant dates.

A prevalence study design derived from the cohort clinical data was used to estimate the one-year period prevalence of the most commonly diagnosed disorders^103^. Sample size calculations estimated that 2,924 Persian cats were needed to represent a disorder with 2% expected prevalence to 0.5% precision at 95% confidence level assuming that Persians comprised 1% from an estimated UK national population of 10 million cats^5,9,104^. This was an observational study only and did not involve any experimentation on live animals. Full ethics approval was obtained from the RVC Ethics and Welfare Committee (URN 2015 1369).

Cats recorded as Persian breed were categorised as Persian (note: these may have included Persian colour variants such as Himalayan [Colourpoint] and Chinchilla); all remaining cats were categorised as non-Persian. ‘All-age bodyweight’ (kg) described all available bodyweight and date combinations. ‘Adult Bodyweight’ described the mean bodyweight recorded from cats aged ≥1 year and was categorised into 4 groups (1.0 to <3 kg, 3.0 to <4.0 kg, 4.0 to <5.0 kg, ≥5.0 kg,). Neuter described the status of the cat (entire or neutered) at the final EPR. Age described the age on December 31^st^ 2013 and was categorised into 6 groups (0.0 to <3.0 years, 3.0 to <6.0 years, 6.0 to <9.0 years, 9.0 to <12.0 years, 12.0 to <15.0 years, ≥15 years).

The sampling frame of unique Persian cats was randomly ordered and the records of all cats reviewed manually to extract the most definitive diagnoses recorded for all disorders that existed during 2013 and to manually link these to the most appropriate VeNom term^78^. Elective (e.g. neutering) or prophylactic (e.g. vaccination) clinical events were not included. No distinction was made between pre-existing and incident disorder presentations. Disorders described within the clinical notes using only presenting sign terms (e.g. ‘vomiting’ or ‘vomiting and diarrhoea’) without a recorded diagnosis term were included using the first clinical sign listed (e.g. vomiting). Mortality data (recorded cause, date and method of death) were extracted on all deaths at any date including after 2013 during the available EPR data.

The extracted diagnosis terms were mapped to a dual hierarchy of precision for analysis: fine-level precision and grouped-level precision as previously described^78^. Briefly, fine-level precision terms described the original extracted terms at the maximal diagnostic precision recorded (e.g. *ulcerative keratitis* would remain as *ulcerative keratitis*). Grouped-level precision terms mapped the original diagnosis terms to a general level of diagnostic precision (e.g. *ulcerative keratitis* would map to *ophthalmological*).

Following data checking and cleaning in Excel (Microsoft Office Excel 2013, Microsoft Corp.), analyses were conducted using Stata Version 13 (Stata Corporation). The sex, neuter status, age and adult bodyweight for the study Persians during 2013 were described. All-age bodyweight data with their associated dates were used to generate individual bodyweight growth curves for male and female Persians by plotting age-specific bodyweights and were overlaid with a cross medians line plot using the Stata *mband* command. One-year period prevalence values were reported along with 95% confidence intervals (CI) that described the probability of diagnosis at least once during 2013. The CI estimates were derived from standard errors based on approximation to the normal distribution for disorders with ≥10 events^105^ or the Wilson approximation method for disorders with <10 events^106^. Prevalence values were reported overall and separately for males and females. The chi-square test was used to compare categorical variables (e.g. male versus female) and Mann-Whitney U test to compare continuous variables (e.g. bodyweight between sexes) as appropriate^105^. Statistical significance was set at the 5% level.

### Ethics approval

This was an observational study only and did not involve any experimentation on live animals. Full ethics approval was obtained from the RVC Ethics and Welfare Committee (URN 2015 1369).

## Data Availability

The datasets generated during and/or analysed during the current study will be made available at the RVC Research Online repository.
